# Table of Urinary Deposits

**Published:** 1855-05

**Authors:** 


We have been furnished, by the author, with a table of Urinary Deposits, with tests Microscopical and Chemical. This is a very useful table, and is the result of much labor and close observation. It will be useful for reference, and particularly valuable to the student. Dr. King, of Cincinnati is the author and deserves much credit for this valuable contribution to medical science.

